# Direct conversion of lignin to high-quality graphene-based materials *via* catalytic carbonization†

**DOI:** 10.1039/d1ra02491d

**Published:** 2021-05-24

**Authors:** Takafumi Ishii, Mikaru Mori, Shiguma Hisayasu, Ryusuke Tamura, Yuki Ikuta, Fumito Fujishiro, Jun-ichi Ozaki, Hideyuki Itabashi, Masanobu Mori

**Affiliations:** International Research and Education Center for Element Science, Faculty of Science and Technology, Gunma University 1-5-1 Tenjin-cho Kiryu Gunma 376-8515 Japan ishii@gunma-u.ac.jp; Faculty of Science and Technology, Kochi University 2-5-1, Akebono-cho Kochi 780-8072 Japan mori@kochi-u.ac.jp; Graduate School of Science and Technology, Kochi University 2-5-1, Akebono-cho Kochi 780-8072 Japan; Graduate School of Science and Technology, Gunma University 1-5-1, Tenjin-cho Kiryu Gunma 376-8515 Japan

## Abstract

Methods for effectively utilizing lignin are necessary for the realization of a sustainable society. Herein, we report a method for directly converting lignin to graphene-based materials. Fe-supported lignin is prepared by dissolving lignin in an aqueous FeCl_2_ solution, followed by freeze drying. Graphene is then produced by catalytically carbonizing this Fe-supported lignin at 1200 °C. The characteristics of both the Fe catalyst and lignin are crucial for the production of high-quality graphene. Specifically, the lignin should disperse well in water, freeze dry, and carbonize *via* solid-state carbonization. The obtained graphene-based material is highly resistant to electrochemical oxidation, as observed in other graphene-based materials. The direct conversion of lignin to graphene described herein is an unprecedented method for synthesizing large amounts of graphene-based material at low cost, as well as being an excellent use for lignin.

## Abstract 2

Directly converting lignin to graphene *via* catalytic carbonization is expected to expand the industrial use of lignin and graphene-based materials.

## Graphical abstract

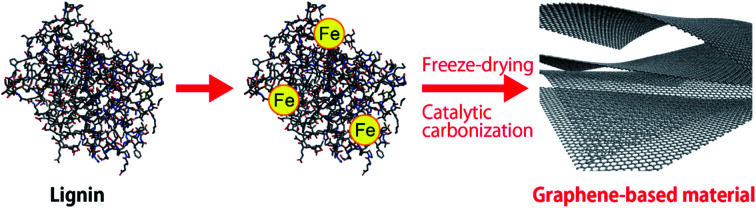

## Introduction

The effective utilization of biomass is expected to contribute toward the realization of a low-carbon society.^1,2^ Carbohydrate-based biomass such as plant-derived cellulose and hemicellulose is used as a raw material for paper, fiber, and bioethanol.^3,4^ The extraction of this biomass leaves large amounts of lignin as a byproduct, a material that has not yet been effectively utilized despite its inexpensive and excessive production.^5^ Various studies on the use of lignin as a precursor to carbon materials have been published.^6–8^ In particular, the applicability of lignin-derived carbon materials in energy storage materials such as capacitors and catalyst materials has been examined.^9,10^

In recent years, various carbon materials have been studied for use in energy storage and conversion devices.^11^ Graphene-based materials have attracted attention as next-generation materials with high specific surface area, chemical stability, and electrical conductivity.^12,13^ In general, graphene-based materials are synthesized from graphene oxide, which is obtained by the chemical treatment of graphite.^14^ However, the synthesis process is complex and has a high environmental impact owing to the use of large amounts of acids and oxidants. Novel methods for the production of graphene using metal catalysts instead of graphene oxide have been proposed. For example, Qiu *et al.*^15^ used chemical vapor deposition (CVD) for the bottom-up deposition of graphene on the surface of a porous metal support, and examined its catalytic applicability. However, this method is not suitable for use in the industrial production of graphene-based materials because of the high price of the raw materials and the difficulty of the CVD process.

To accelerate the industrial use of graphene-based materials, it is necessary to develop a cheap, environmentally friendly, and sustainable synthetic method that does not use graphene oxide. In this study, we report a new method for synthesizing graphene-based material directly from lignin. In recent years, several studies have been reported on methods for producing graphene-based materials using lignin as a raw material, but these methods have not yet led to the synthesis of superior graphene (*i.e.*, high graphene content and few defect sites).^16,17^ The proposed method utilizes the water solubility and ion exchange capacity of lignin to produce superior graphene directly by catalytic carbonization. The direct conversion from lignin to graphene is sustainable, inexpensive, and would provide large amounts of graphene-based material. Moreover, it is expected to greatly contribute to the industrial use of both lignin and graphene-based materials.

## Experimental section

Commercially available alkaline lignin (L0082, Tokyo Chemical Industry Co., Ltd.) was used as the lignin sample. The lignin sample (1.0 g) was dispersed in FeCl_2_ solution (50 mL) at a predetermined concentration, and the solution was stirred for 3 h. To prevent the oxidation and precipitation of Fe^2+^, hydroxylammonium chloride and acetic acid were added to the FeCl_2_ solution at respective molar ratios of 0.36 and 0.007 (for 1 mol of Fe^2+^). Lignin samples supported with Fe (Fe-supported lignin) were prepared by oven drying or freeze drying the obtained aqueous lignin solutions. The Fe loading was adjusted in the range of 0.028 to 2.8 mmol g^−1^. The Fe-supported lignin samples were heat-treated for 1 h in an inert atmosphere at a temperature of 1200 °C to obtain carbonized samples. The sample names are distinguished by the amount of Fe (*X* mmol g^−1^) and the lignin drying method (oven dried (OD) or freeze dried (FD)), and are expressed as L-Fe*X*(OD) and L-Fe*X*(FD). As control samples, metal-free carbonized samples were prepared by dispersing lignin in distilled water without FeCl_2_ followed by drying (OD or FD) and heat treating under the conditions described above. The control samples are denoted as L(OD) and L(FD), respectively.

Carbonized samples were observed using transmission electron microscopy (TEM, JEM-2010, JEOL Co.) to investigate their carbon structures. Sample lattice fringes on the microgrid were analyzed at an accelerating voltage of 200 kV. The surface areas and pore structures were evaluated using nitrogen adsorption–desorption measurements (BELSORP mini, MicrotracBEL Co.) at −196 °C. The specific surface areas were calculated based on Brunauer–Emmett–Teller (BET) theory. The volumes of mesopores (*V*_meso_) were determined by Barrett–Joyner–Halenda (BJH) analysis. Raman spectra of the prepared carbon samples were obtained using Raman spectrometry (Nicolet Almega XR, Thermo Fisher Scientific, Inc.) with a 532 nm Nd:YAG laser. X-ray diffraction (XRD) analysis was performed by an X-ray diffractometer (XRD-6100, Shimadzu Co.) with Cu Kα radiation. X-ray photoelectron spectroscopy (XPS) measurements were performed with a Kratos AXIS NOVA instrument (Shimadzu Co.). The charge-up shift correction was conducted by setting the C 1s binding energy level of the samples to 284.5 eV.

Temperature-programmed desorption mass spectrometry (TPD-MS) was used to measure the concentrations of the peripheral (edge) sites of the graphene layers in the carbonized samples by measuring the molecules desorbing at those sites. The average size of the graphene sheet was also calculated from the number of edge sites.^18^ A detailed description of the experimental setup and operation is provided elsewhere.^19,20^

Electrochemical measurements were performed using a disk electrode. The working electrode was prepared by covering a glass-like carbon disk electrode with a mixture of the prepared carbonized sample (200 μg cm^−2^) and Nafion®. To evaluate the electrochemical oxidation resistance and compare the electrochemical characteristics with other graphene-based materials, cyclic voltammetry (CV) was performed in the following manner. First, a CV scan (5 mV s^−1^) was repeated four times in the potential range of 0 to 1.0 V *vs.* a reversible hydrogen electrode (RHE) in 0.5 M H_2_SO_4_ aqueous solution at room temperature. Then, triangle-wave potential cycling between 1.0 and 1.5 V at 100 mV s^−1^ was employed as a carbon oxidation test. After 100, 200, and 500 cycles, the CV scan was repeated another four times in the potential range of 0 to 1.0 V.

## Results and discussion

**Fig. 1:**
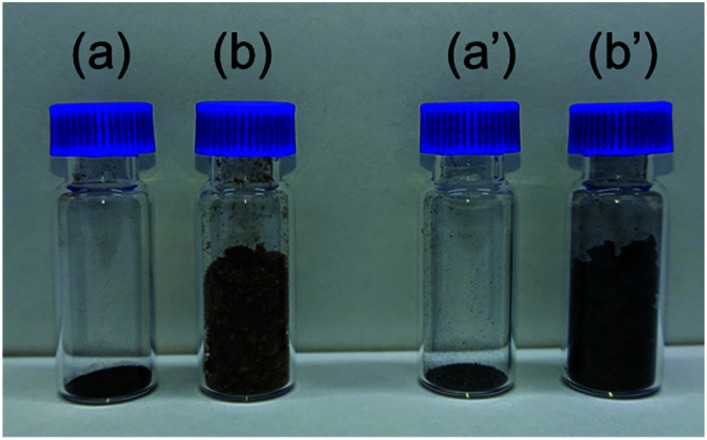
Photographs of (a, b) lignin samples and (a′, b′) carbonized lignin samples obtained by (a) oven drying and (b) freeze drying of lignin. Each sample was prepared from the same amount of lignin.

Lignin molecules possess many oxygen-containing functional groups.^21^ In addition, the alkaline lignin used in this study was water-soluble. Therefore, the morphology of the obtained lignin sample differs greatly depending on the drying method of the lignin solution. Fig. 1a and b show lignin samples obtained by oven drying and freeze drying, respectively. The oven-dried samples were dense powders, with characteristics similar to solid resin. In contrast, freeze-drying yielded fluffy powders. Fig. 1a′ and b′ show the carbonized samples prepared by heat treating the oven- and freeze-dried lignin samples at 1200 °C. For both drying methods, the powder texture was maintained after heat treatment. Lignin is a polymer with characteristics comparable to thermosetting resins,^22^ which are generally converted to carbon by solid-phase carbonization.^23^ In solid-phase carbonization, the polymer is carbonized without melting; therefore, the lignin samples maintain their powder shape. The morphology and carbonization behavior of lignin are strongly related to the formation of graphene, as described later.

**Fig. 2:**
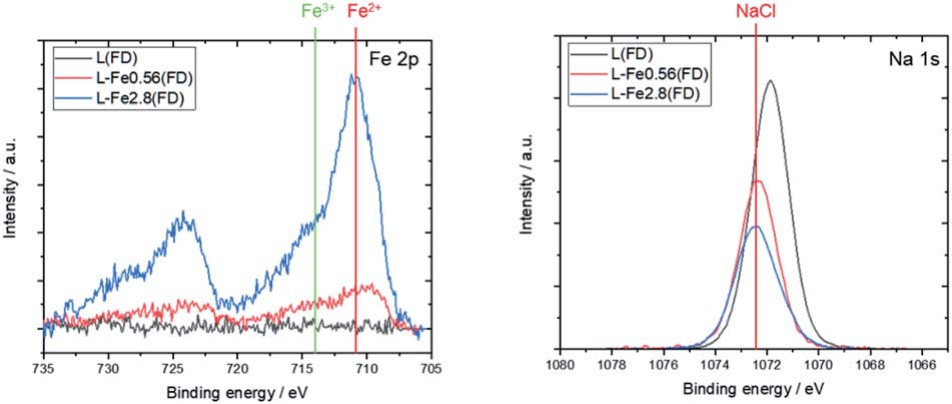
XPS spectra of L(FD), L-Fe0.56(FD), and L-Fe2.8(FD) before the heat treatment. The positions of Fe^2+^, Fe^3+^, and NaCl are taken from previous studies.^24,25^

Fig. 2 shows the XPS spectra of the freeze-dried lignin samples with different Fe loading. The Fe 2p spectrum indicates that the Fe, loaded on the lignin, is in Fe^2+^ state. The Na 1s peaks are shifted toward the higher energy side due to Fe loading. This shift can be attributed to the formation of NaCl, thereby indicating that Na in the lignin was converted to NaCl as a result of Fe loading. In this study, FeCl_2_ was used as the Fe source, and Na, coordinated to the oxygen-containing functional group in lignin, was ion exchanged with Fe^2+^, resulting in the formation of NaCl. This suggests that the Fe that is supported on the lignin is coordinated to the oxygen-containing functional groups in the lignin as Fe^2+^.

**Fig. 3:**
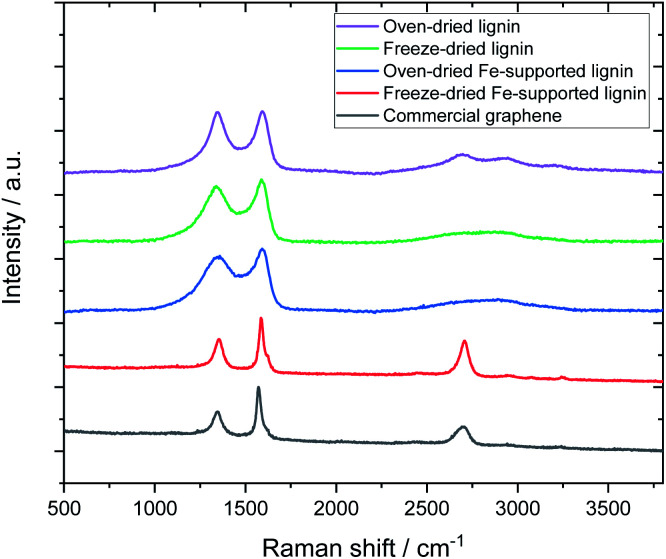
Effect of drying method and Fe support on Raman spectra of carbonized samples.

Fig. 3 shows the Raman spectra of the carbonized samples. For comparison, the Raman spectrum of commercially available graphene is also shown. For the control (metal-free, carbonized) samples, broad Raman spectra were confirmed regardless of the drying method, which is a common characteristic of amorphous carbon.^26,27^ The oven-dried Fe-supported lignin sample (L-Fe0.56(OD)) exhibited a similar Raman spectrum to the metal-free sample, whereas the freeze-dried Fe-supported sample (L-Fe0.56(FD)) showed a strong 2D band (2700 cm^−1^) unique to graphene.^28^ Notably, the Raman spectrum of L-Fe0.56(FD) is very similar to that of commercially available graphene (G0500, Tokyo Chemical Industry Co., Ltd.). Attempts to synthesize graphene from lignin have been reported so far, but the graphene obtained in those reports showed a relatively weak 2D peak.^16,17^ L-Fe0.56(FD) obtained by our method shows a stronger 2D band than not only the reported graphene but also the reduced graphene oxide which is often used in the production of graphene-based materials. There has never been a report of such high-quality graphene being directly synthesized from lignin, which indicates the usefulness of our approach as a method for converting lignin to graphene.

**Fig. 4:**
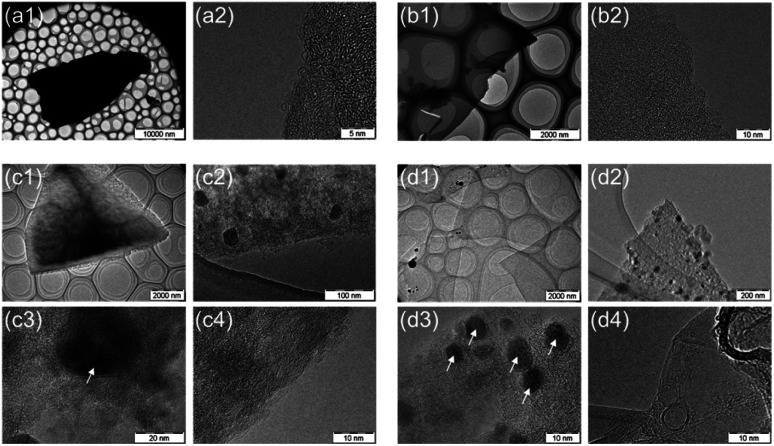
TEM images of carbonized samples obtained from (a1, a2) oven-dried lignin (L(OD)), (b1, b2) freeze-dried lignin (L(FD)), (c1–c4) oven-dried Fe-supported lignin (L-Fe0.56(OD)) and (d1–d4) freeze-dried Fe-supported lignin (L-Fe0.56(FD)), respectively. The white arrows in the figures indicate the metal particles in the carbon structures.

Fig. 4 shows TEM images of the carbonized sample. The oven-dried samples have angular, powdered shapes, whereas the freeze-dried samples possess huge sheet-like shapes. The macroscopic structure differs depending on the drying method. Focusing on the micro carbon structure, thin graphene-like carbon films were observed only in L-Fe0.56(FD), whereas the other samples showed an amorphous carbon structure, which indicates that both Fe support and freeze drying play a major role in producing graphene from lignin. The TEM images of the Fe-containing samples showed the existence of metallic particles with sizes ranging from several nanometers to several tens of nanometers. Furthermore, these particles were composed of two components: metallic Fe and Fe_3_O_4_, as verified by XRD measurements and selected area electron diffraction (SAED) analysis (Fig. S1 and S2†).

**Fig. 5:**
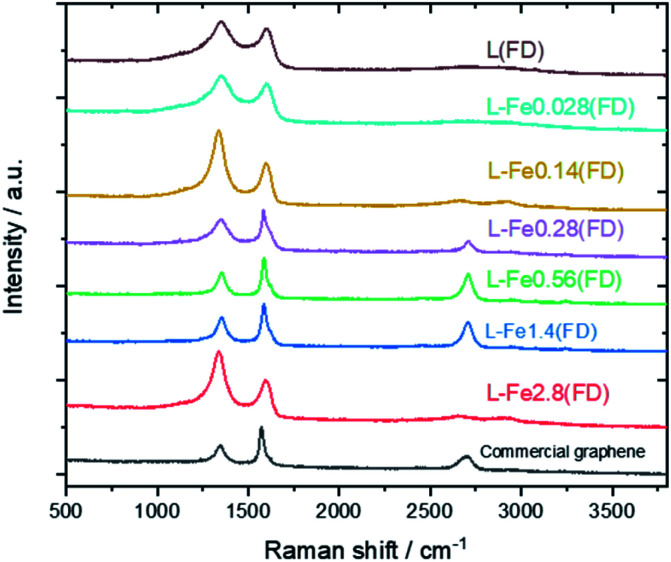
Raman spectra of carbonized lignin samples prepared from freeze-dried lignin with different Fe loadings.

**Fig. 6:**
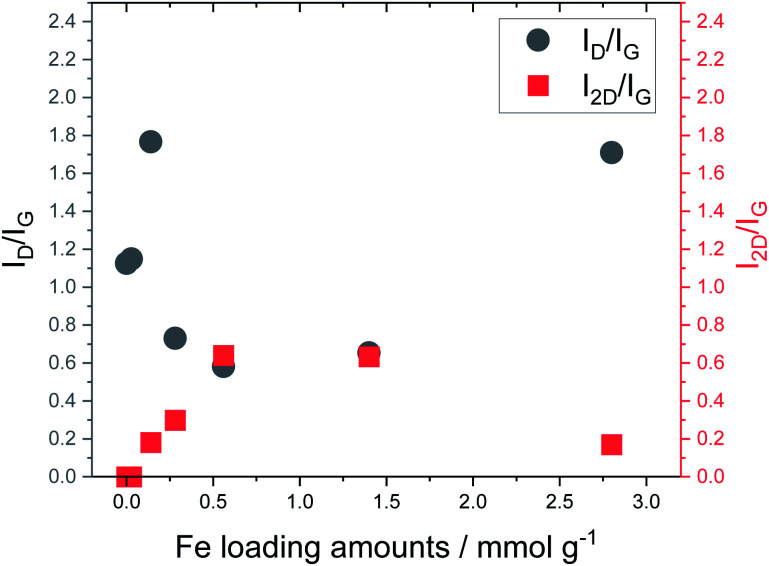
Relationship between Fe loading and *I*_D_/*I*_G_ and *I*_2D_/*I*_G_ ratios calculated from Raman spectra of carbonized lignin samples.

Fig. 5 shows the Raman spectra of carbonized lignin samples prepared from freeze-dried lignin with different Fe loadings. Graphene formation was confirmed at approximately 0.56 mmol g^−1^ of Fe. Interestingly, the intensity of the graphene-specific 2D band was greatly reduced when the metal loading was increased to 2.8 mmol g^−1^, indicating that graphene was not produced. To quantitatively discuss the formation of graphene, the intensity ratios of the D band (1350 cm^−1^) to the G band (1590 cm^−1^) (*I*_D_/*I*_G_) and the 2D band to the G band (*I*_2D_/*I*_G_) were calculated. The results are summarized in Fig. 6. *I*_D_/*I*_G_ was minimized, and *I*_2D_/*I*_G_ was maximized, with between 0.28 and 1.4 mmol g^−1^ of metal loading. When the lignin contained 2.8 mmol g^−1^ of Fe, the *I*_D_/*I*_G_ and *I*_2D_/*I*_G_ values were almost the same as those of the control sample (metal-free carbonized lignin), which indicates that the beneficial effect of the Fe support on the production of graphene is lost at a high metal loading.

**Fig. 7:**
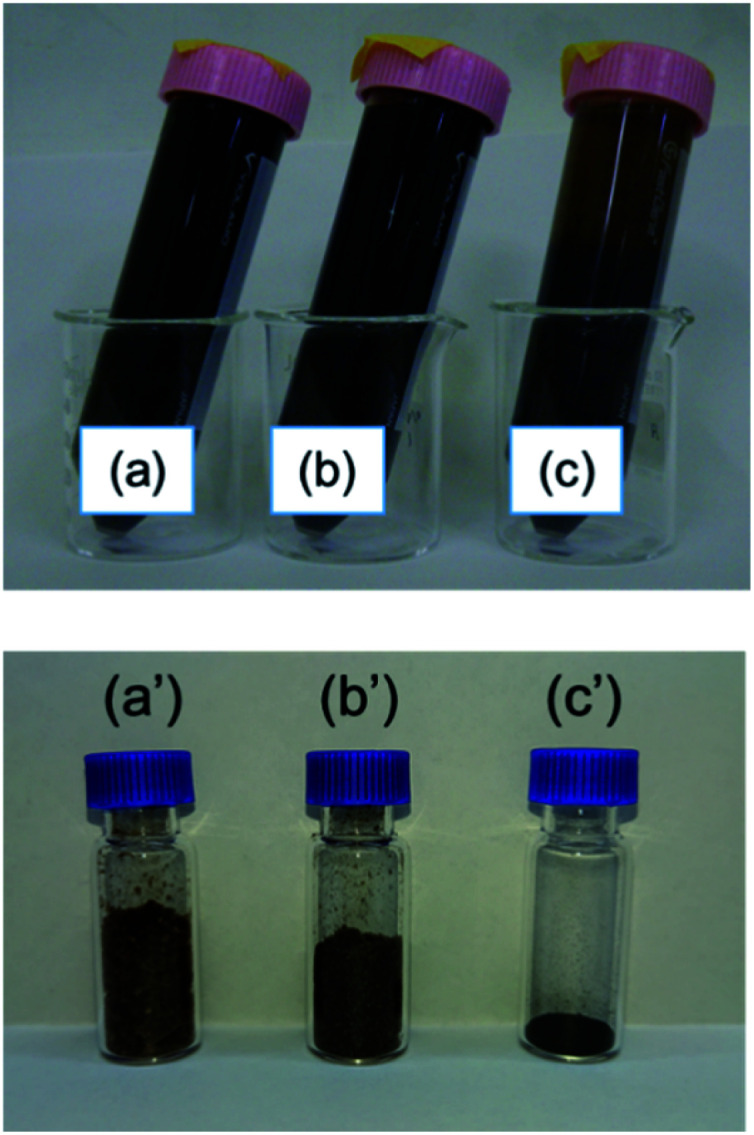
Photographs of (a–c) dispersed solutions and (a′–c′) freeze-dried lignin containing (a, a′) lignin (b, b′) 0.56 mmol g^−1^ Fe-supported lignin, and (c, c′) 2.8 mmol g^−1^ Fe-supported lignin.

Fig. 7 shows photographs of the lignin solutions and freeze-dried lignin samples with different Fe loadings. Lignin precipitates from the solution when the metal loading is increased to 2.8 mmol g^−1^ because lignin cannot maintain its dispersed state in an aqueous solution with a large amount of metal cations.^29^ The presence of this precipitate causes the morphology of the freeze-dried sample to differ from that of the other freeze-dried samples; the apparent density is clearly reduced, and the state appears similar to that of the oven-dried sample. This indicates that two factors are essential for graphene production from lignin, namely, solidification of the lignin molecules in a sparse state, and appropriate Fe support for the lignin molecules.

Since graphene aggregates are easily laminated by van der Waals forces, the strong Raman 2D band is unique to graphene; it cannot be observed in general carbon materials. To prevent the lamination of graphene, it is considered appropriate to use a carbon precursor in which the polymer is spatially sparse, such as freeze-dried lignin. So far, we have reported on the catalytic and noncatalytic carbonization of polymers,^30–33^ but the formation of graphene observed herein has not been reported previously. Hydrophobic polymers are generally used as carbon precursors in carbon chemistry and industry. Such polymers are difficult to freeze dry, and when used as carbon precursors, a dense solid powder must be used. It is conceivable that a dense carbon precursor would yield dense carbon, that is, carbon with laminated graphene. In addition to the effect of the Fe catalyst, the use of a sparse polymer at the molecular level—such as freeze-dried lignin—is considered the most important factor in the formation of graphene in this method.

To clarify the principle of graphene formation from metal-supported lignin, we performed a detailed structural analysis of the carbonized samples. Table 1 shows the average size of the graphene sheets calculated from TPD measurements and the BET specific surface area and mesopore volume of each carbonized sample. Carbonized L(OD) and L(FD) were prepared from oven-dried and freeze-dried metal-free lignin solutions, respectively. Although the presence of pores could not be confirmed in the L(OD) sample, the L(FD) sample had a pore structure with some mesopores. By freeze drying, the lignin molecules are solidified in a spatially dilute state. The subsequent solid-state carbonization process maintains the spatial arrangement of the lignin molecules to some extent, yielding the observed pore structure. Although the difference in the drying method greatly affects the pore structure of the obtained carbon, the carbonized L(OD) and L(FD) samples had a similar graphene sheet size, which indicates that the growth of the graphene sheets is almost the same during carbonization.

**Table 1:** Properties of carbonized lignin samples

Sample	BET specific surface area^aa^ (m^2^ g^−1^)	*V*_meso_ aa ^,^ bb (cm^3^ g^−1^)	Total edge sites^cc^ (μmol g^−1^)	Average size of graphene sheets^c^ (nm)
L(OD)	<1	N/A	698 ± 79	60 ± 6
L(FD)	312	0.019	853 ± 101	49 ± 5
L-Fe0.56(FD)	440	0.084	457 ± 124	97 ± 26
L-Fe2.8(FD)	330	0.085	721 ± 178	61 ± 15

a Determined by N_2_ adsorption isotherm.

b Volume of mesopores.

c Obtained from TPD-MS.

In our previous report,^18^ we found that the graphene sheet sizes of carbon materials had a greater dependence on the temperature of carbonization than on the carbon structure or pore structure. By comparing previous results with the current report, it can be interpreted that the spatial distribution of lignin molecules does not affect the growth rate of graphene sheets. In contrast, the sheet size of L-Fe0.56(FD), in which graphene formation was confirmed, was clearly larger than that of the other samples. This was due to the presence of the Fe support, which causes a catalytic carbonization reaction and promotes the growth of the graphene sheet. The sheet size of L-Fe2.8(FD), which had a large Fe^2+^ loading, was approximately the same as that of L(FD), which was formed without Fe^2+^. Therefore, the effect of catalytic carbonization cannot be confirmed for L-Fe2.8(FD). L-Fe0.56(FD) and L-Fe2.8(FD) differed in whether they exhibited lignin aggregation, as shown in Fig. 7, as well as in the amount of supported Fe^2+^.

To proceed successfully with the catalytic carbonization reaction, optimal Fe loading and spatial distribution of lignin molecules are required. When the heat treatment temperature was reduced to 1100 °C or lower (data not shown), graphene formation was not confirmed for any of the lignin samples. The catalytic carbonization reaction is promoted as the temperature increases. With Fe catalysts, the catalytic carbonization reaction should start at approximately 700 °C.^31^ However, the catalytic carbonization of lignin herein proceeded at an extremely high temperature of 1200 °C, which differs from previous reports on catalytic carbonization. To understand the catalytic carbonization of lignin, it is necessary to conduct more detailed investigations in the future. The high water dispersibility and solid-state carbonization characteristics of lignin play important roles in the formation of graphene from lignin. The direct conversion of lignin to graphene by catalytic carbonization in this study was achieved owing to the polymer properties of lignin.

**Fig. 8:**
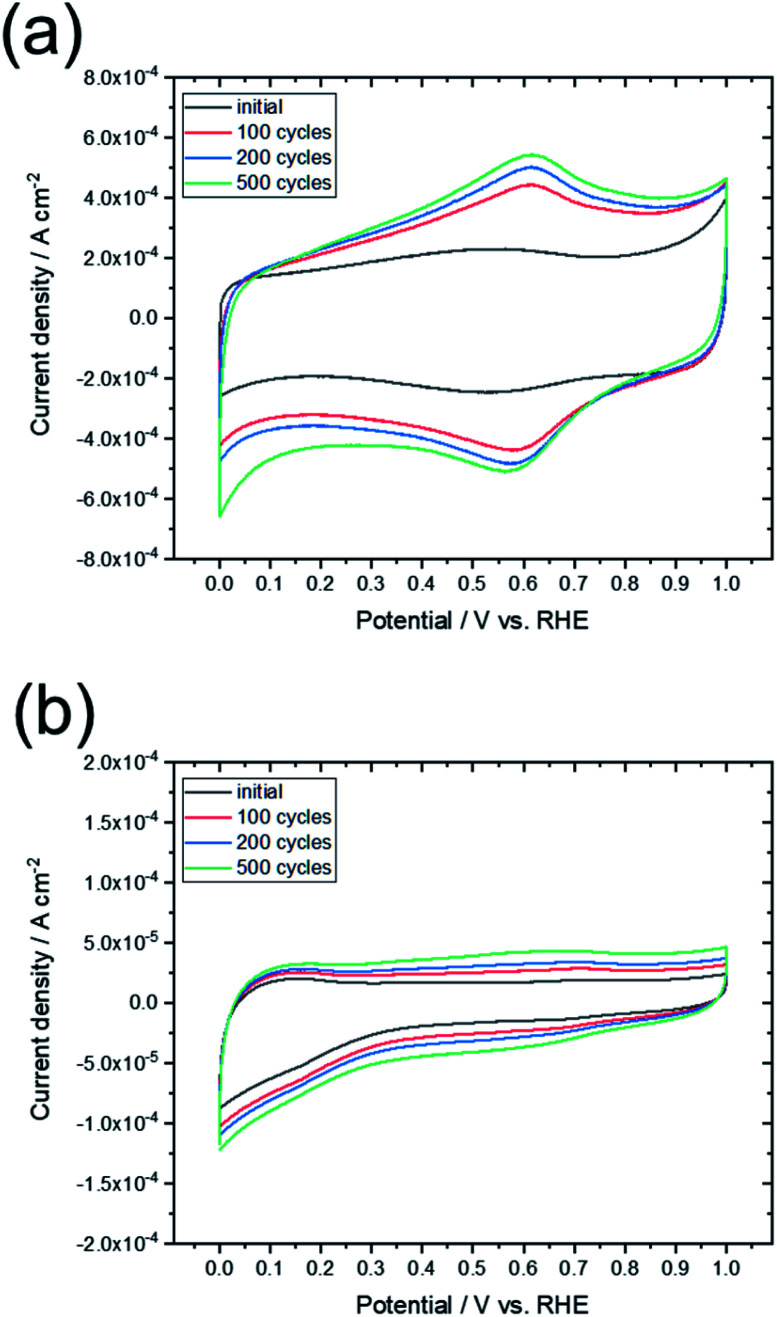
CV curves obtained before and after the oxidation cycling tests for (a) commercially available activated carbon (YP50F) and (b) L-Fe0.56(FD).

To compare the characteristics of the obtained graphene-based material with those of other graphene-based materials, the sample was electrochemically evaluated by CV and oxidation cycling. Fig. 8 shows the CV curves of L-Fe0.56(FD) and a commercially available activated carbon (YP50F, Kuraray Chemical. Co.). The CV curve for activated carbon changed significantly upon oxidation cycling, which indicates that many surface functional groups were formed by oxidation of the carbon. In contrast, the shape of the CV curve for L-Fe0.56(FD) was maintained even as oxidation cycling continued. The electrochemical oxidation of carbon materials proceeds from the carbon edge sites, which are typical defect sites in graphene. As shown in Table 1, the graphene sheet size of L-Fe0.56(FD) is much larger than that of YP50F (7–8 nm, determined from TPD measurements). This indicates that the L-Fe0.56(FD) sample has few carbon edge sites. The formation of graphene from lignin *via* catalytic carbonization promotes the reduction of these carbon edge sites, thereby improving the electrochemical oxidation resistance. Resistance to electrochemical oxidation is a common characteristic of graphene-based materials.^13^ Thus, this result indicates that the carbonized sample can be regarded as a graphene-based material.

## Conclusion

In this study, we show that graphene can be synthesized from lignin biomass, which is abundant in modern society. To generate graphene from lignin, two essential conditions were identified: (i) the metal-supported lignin must remain well-dispersed in an aqueous solution, and (ii) a low-density powder is required, such as that produced by freeze-drying. The water dispersibility and solid-state carbonization characteristics of lignin play important roles in conversion to graphene. Lignin-derived graphene exhibited similar electrochemical properties to other graphene-based materials. The direct conversion of lignin to graphene is an unprecedented method for synthesizing large amounts of graphene-based material at low cost and is also an excellent method for the effective use of excessive lignin produced when extracting cellulose. With further research, this method for producing graphene-based materials could revolutionize the sustainability of the energy we use.

## Author contributions

The manuscript was written through contributions of all authors. All authors have given approval to the final version of the manuscript.

## Conflicts of interest

There are no conflicts to declare.

## Supplementary Material

RA-011-D1RA02491D-s001
