# Supplementary material for: Direct conversion of lignin to high-quality graphene-based materials via catalytic carbonization
Source: RSC Adv. 2021 May 24;11(31):18702–7. doi: 10.1039/d1ra02491d (PMC9033452; doi:10.1039/d1ra02491d)
Supplement: RA-011-D1RA02491D-s001 [file RA-011-D1RA02491D-s001.pdf]

Electronic Supplementary Material (ESI) for RSC Advances.
This journal is © The Royal Society of Chemistry 2021

Direct conversion of lignin to high-quality graphene-based materials via catalytic carbonization

Takafumi Ishii[a,*], Mikaru Mori[b], Shiguma Hisayasu[c], Ryusuke Tamura[c], Yuki Ikuta[c], Fumito Fujishiro[b,c], Jun-ichi Ozaki[a], Hideyuki Itabashi[d], Masanobu Mori[b,c,*]

Supporting Information

*Corresponding author: Takafumi Ishii, Dr.

Telephone number: +81-277-30-1350

E-mail address: ishii@gunma-u.ac.jp

*Corresponding author: Masanobu Mori, Dr.

Telephone number: +81-88-844-8306

E-mail address: mori@kochi-u.ac.jp

[a] *International Research and Education Center for Element Science, Faculty of Science and Technology, Gunma University, 1-5-1 Tenjin-cho, Kiryu, Gunma 376-8515, Japan.*
[b] *Faculty of Science and Technology, Kochi University, 2-5-1, Akebono-cho, Kochi 780-8072, Japan.*
[c] *Graduate School of Science and Technology, Kochi University, 2-5-1, Akebono-cho, Kochi 780-8072, Japan.*
[d] *Graduate School of Science and Technology, Gunma University, 1-5-1, Tenjin-cho, Kiryu, Gunma 376-8515, Japan*

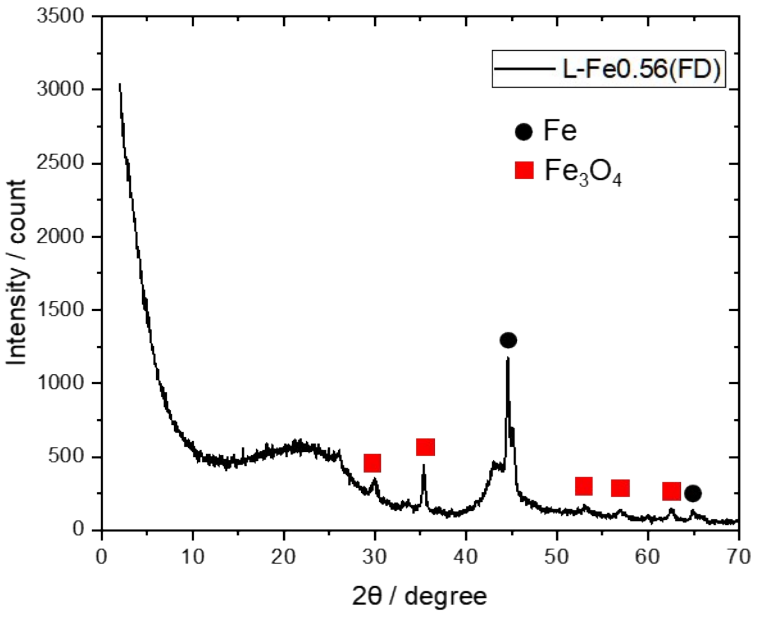

Figure S1. XRD pattern of L-Fe0.56(FD).

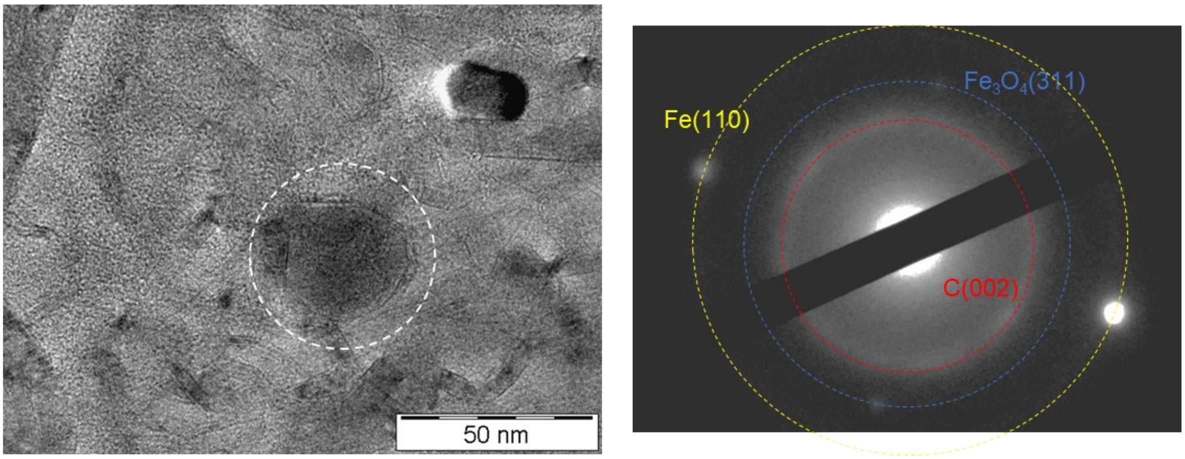

Figure S2. TEM image and SAED pattern of L-Fe0.56(FD). The SAED pattern was obtained from the region indicated by the white circle in the TEM image.
